# Automated Classification of 6-n-Propylthiouracil Taster Status with Machine Learning

**DOI:** 10.3390/nu14020252

**Published:** 2022-01-07

**Authors:** Lala Chaimae Naciri, Mariano Mastinu, Roberto Crnjar, Iole Tomassini Barbarossa, Melania Melis

**Affiliations:** Department of Biomedical Sciences, University of Cagliari, Monserrato, 09042 Cagliari, Italy; lala.chaimae.naciri@gmail.com (L.C.N.); mariano.mastinu@unica.it (M.M.); crnjar@unica.it (R.C.); melaniamelis@unica.it (M.M.)

**Keywords:** PROP taster status, ratings of perceived taste intensity, papilla density, *TAS2R38*, supervised learning (SL)

## Abstract

Several studies have used taste sensitivity to 6-n-propylthiouracil (PROP) to evaluate interindividual taste variability and its impact on food preferences, nutrition, and health. We used a supervised learning (SL) approach for the automatic identification of the PROP taster categories (super taster (ST); medium taster (MT); and non-taster (NT)) of 84 subjects (aged 18–40 years). Biological features determined from subjects were included for the training system. Results showed that SL enables the automatic identification of objective PROP taster status, with high precision (97%). The biological features were classified in order of importance in facilitating learning and as prediction factors. The ratings of perceived taste intensity for PROP paper disks (50 mM) and PROP solution (3.2 mM), along with fungiform papilla density, were the most important features, and high estimated values pushed toward ST prediction, while low values leaned toward NT prediction. Furthermore, *TAS2R38* genotypes were significant features (AVI/AVI, PAV/PAV, and PAV/AVI to classify NTs, STs, and MTs, respectively). These results, in showing that the SL approach enables an automatic, immediate, scalable, and high-precision classification of PROP taster status, suggest that it may represent an objective and reliable tool in taste physiology studies, with applications ranging from basic science and medicine to food sciences.

## 1. Introduction

Taste is the sensory modality that enables organisms to distinguish nutrient-rich food from harmful substances [1,2]; it recognizes five basic sensory qualities: sweet, salty, sour, bitter, and umami. It is well known that the ability to perceive a taste varies greatly between individuals, and that the same kind of food can taste very different to different individuals [3]. Therefore, the sense of taste can significantly influence the food choices, nutrition, and health of the individual [4]. Several physiological studies have focused on the ability to taste the bitter compound 6-n-propylthiouracil (PROP), with the aim of evaluating the individual variability of taste perception in humans [4,5,6,7,8]. Individuals can be classified as belonging to one of the three PROP taster categories (PROP non-taster (NT), medium taster (MT), and super taster (ST)), based on the determination of their PROP sensitivity, by using several psychophysical methods [4,5,6,7,9]. PROP taste detection is mediated by the *TAS2R38* bitter receptor, and the individual variability in the ability to perceive this stimulus is greatly associated with haplotypes of the *TAS2R38* gene [10,11]. There are two common haplotypes: the PAV variant, which has high affinity for PROP, is dominant, while AVI, the recessive variant, has low or null affinity for PROP. NTs are almost always homozygous for the AVI haplotype, while STs have been assumed to be homozygous for the PAV haplotype, and MTs are heterozygous [12]. However, some studies have reported a considerable genotypic overlap between the MTs and STs [10,13], while others have shown that the presence of two PAV haplotypes (as opposed to one) confers no additional benefit for perceiving more bitterness from PROP [8]. Therefore, the haplotypic diversity in the *TAS2R38* gene does not completely explain the differences in the ability to taste PROP, thus implying that other factors may be involved [4,8,11,14], such as the density and function of the fungiform papillae [15]. Several studies have consistently reported that STs have a higher density of fungiform taste papillae [6,16,17,18] or a greater functional activity [15] compared to the other PROP taster groups. These morphological features of STs can explain why these individuals are more responsive to a wide range of oral stimuli that are not mediated via the *TAS2R38* bitter receptor [9,19,20,21,22,23,24,25,26,27,28,29,30,31,32]. Some authors have shown that a polymorphism in the gene codifying for gustin—the salivary taste bud trophic factor—is associated with differences in the fungiform papilla density and function, and with differences in the chemosensory ability across PROP phenotypic groups [15,33,34]. However, other studies have failed to find associations between PROP tasting and fungiform papilla density and/or gustin genotypes [35,36]. Oral sensitivity to PROP has also been related to other modifying genes [37,38], or to levels of specific amino acids and proteins in saliva [39,40,41]. Several studies have reported gender differences in PROP perception, showing that women were more sensitive to PROP than men [6,42,43], and had more taste buds and fungiform papillae [6,44]; however, other studies do not substantiate these differences [15,45,46].

In recent decades, great attention has been paid to PROP tasting as an oral biomarker of general taste perception, affecting food preferences, eating behaviors, nutritional status, and health [3,4,47]. Several studies have reported that subjects who perceive PROP as intensely bitter (PROP STs) have a higher sensitivity than NTs to various taste stimuli that are not mediated via the specific receptor, including other bitter chemicals [19,20,21,48,49,50], sweet stimuli [25], sour compounds [23], umami taste [26], etc. Several studies have shown relationships between PROP phenotype or genotype and longevity [51], age [52,53], or a number of health parameters, including antioxidant status [54], body mass index (BMI) [13,55,56,57], metabolic changes [58,59], smoking status [60,61,62], alcohol consumption [24], respiratory infections [63,64], taste disorders [65], colonic neoplasm risk [66,67], and neurodegenerative diseases [68]. However, the validity of PROP tasting as a biomarker has been questioned by some authors, who have shown inconsistent results [58,59,69,70,71,72,73,74,75,76,77,78]. The major issues that have led to these controversial results are the differential characteristics of the studied populations, of the methods used to assess health parameters, or of the psychophysical approaches used to identify the PROP phenotype which, being based on highly subjective evaluations, can produce significant measurement errors [35].

The main aim of this work was to automatically classify subjects as belonging to the three PROP taster categories by using machine learning (ML) [79] that provides real-time decision making, which could make this process immediate and scalable. This paper addresses for the first time the problem of evaluating the effectiveness of ML classifiers in the automatic discrimination between subjects belonging to the PROP taster categories (assigned to the subjects by standard non-objective scaling methods), by exploiting the biological features of the subjects. Furthermore, the proposed model was intended for understanding of the importance and the impact of each biological feature on the PROP taster status of subjects, thus also enabling the validation of a single marker for its determination.

The biological features used as predictive variables were sensory, genetic, morphological, clinical, and demographic data, which are regularly used for the classification of PROP taster status in psychophysical approaches (e.g., taste intensity ratings for PROP and NaCl [5,7]). or have been associated with PROP taster status in a large number of studies (e.g., density of fungiform papillae and genotypes of *TAS2R38* and CA6 genes, age, gender, BMI, scores for taste quality sensitivity, and smoking status [3,4,6,10,11,13,15,55,56,60,61,62,80,81]). 

## 2. Materials and Methods

### 2.1. Study Design

The automatic classification of subjects as belonging to the three PROP taster categories was carried out using ML [79]. ML uses algorithms and computational statistics to learn from data without explicitly computing them. Among the three approaches of ML (reinforcement learning (RL); unsupervised learning (UL); and supervised learning (SL)), we used the supervised classifier method by applying different algorithms and assembling a structured dataset consisting of the biological features of subjects that were used as inputs for the algorithms. Specifically, the SL algorithm classifiers used the labeled dataset to learn and create a classification model that evaluated the differences between subjects and returned a high-precision prediction on the PROP taster status of the subject. 

**Figure 1:**
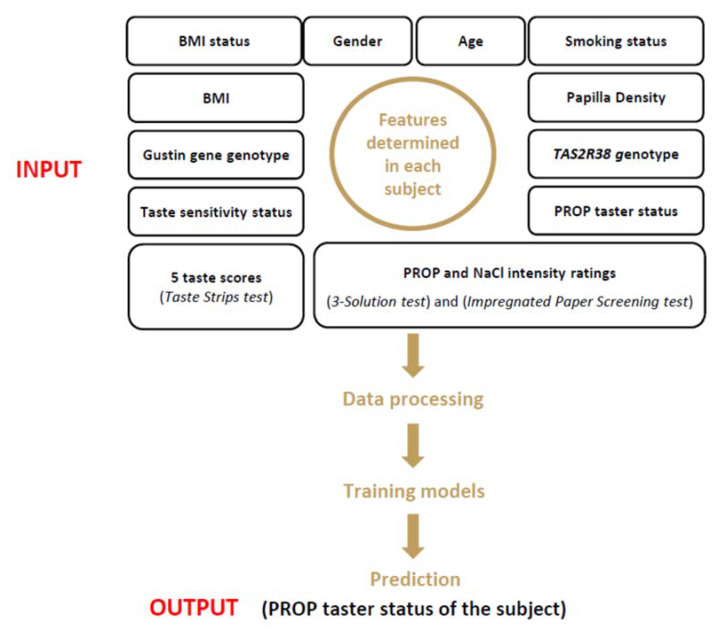
Graphic diagram representing the study design.

The overall design of the study is depicted in Figure 1.

### 2.2. Experimental Procedure

For each subject, the following biological data were determined: PROP and NaCl intensity ratings, five taste scores, papilla density, BMI, and age (which were classified as numerical data), as well as *TAS2R38* genotypes, gustin gene genotypes, taste sensitivity status, BMI status, smoking status, and gender (which were classified as categorical data). 

Data were collected for each subject in two sessions on two successive days. For sensory analyses, subjects were requested to abstain from drinking (except water), eating, and using chewing gum or oral care products for at least 2 h prior to testing. All subjects had to be in the test room 15 min before the beginning of the session in order to adapt to the environmental conditions (23–24 °C; 40–50% relative humidity). Women were tested on the sixth/seventh day of their menstrual cycles in order to avoid taste sensitivity changes due to the estrogen phase [82]. 

In the first session, weight (kg) and height (m) were recorded in order to calculate the subjects’ BMI (kg/m^2^). Taste intensity ratings for PROP and NaCl were collected by using two validated psychophysical approaches (the three-solution test [5], and the filter paper method [7]), and samples of the whole saliva (2 mL) were collected and stored at −80 °C until the molecular analyses were completed.

In the second session, taste sensitivity to the five primary qualities (sweet, sour, salty, bitter, and umami) was examined by using the taste strip test (TST; Burghart Messtechnik, Wedel, Germany) [83,84] and umami test (Burghart Messtechnik, Wedel, Germany) [85]. The density of the fungiform papillae was also measured. 

All taste solutions were previously prepared, stored in a refrigerator, and presented for the sensory measures at room temperature.

### 2.3. Subjects

A total of 84 Caucasian subjects (49 women and 35 men) were recruited through usual procedures at the University of Cagliari, Italy; all were originally from the island of Sardinia, Italy. Mean age was 25.07 ± 0.507 y, ranging from 18 to 40 y; 19 subjects were smokers and 65 were non-smokers. Subjects were classified as underweight (*n* = 10), normal weight (*n* = 58), or overweight (*n* = 16) based on their BMI: underweight subjects had a BMI below 18.5 kg/m^2^, normal-weight subjects from 18.5 to 24.9 kg/m^2^, and overweight subjects from 25.0 to 29.9 kg/m^2^. 

Exclusion criteria were major systemic diseases, use of drugs interfering with taste or smell (e.g., steroids, antihistamines, and certain antidepressants), pregnancy or lactation, and food allergies. All subjects provided a signed informed consent form prior to being enrolled in the study. The study was conducted according to the guidelines of the Declaration of Helsinki, and approved by the Ethical Committee of the University Hospital of Cagliari (protocol code: 451/09; date of approval: 5/2016).

### 2.4. Sensory Assessments

#### 2.4.1. PROP and NaCl Intensity Ratings 

The three-solution test, [5] and the impregnated paper screening test [7], which were used to collect PROP and NaCl intensity ratings, allowed us to classify subjects based on their PROP bitter taste status. These two methods have been used in numerous studies [33,34,40,81], and are strongly correlated with the degree of activation of peripheral taste function [86,87,88].

Subjects were first assessed using the three-solution test, as described by Tepper et al. [5]. The perceived intensity ratings of three suprathreshold solutions of sodium chloride (NaCl; 0.01, 0.1, and 1.0 mol/L) (Sigma-Aldrich, Milan, Italy) and of PROP (0.032, 0.32, and 3.2 mmol/L) (Sigma-Aldrich) were collected by using the labeled magnitude scale (LMS) [89] in each subject. The LMS allows subjects to rate the intensity of PROP bitterness relative to the “strongest imaginable” oral stimulus that they have ever experienced in their life. Concentrations (10 mL samples) of each solution type were presented in a random order. Each stimulus was followed by oral rinsing with spring water, and the interstimulus interval was set at 60 s. Subjects who gave lower ratings to PROP than to NaCl were classified as NTs, those who gave overlapping ratings to the two chemicals were classified as MTs, and those who gave higher ratings to PROP than to NaCl were classified as STs. After a 1-hour period, the belonging of each subject to a PROP taster category was validated by using the impregnated paper screening test [7]. PROP (50 mmol/L) and NaCl (1.0 mol/L) were presented via two impregnated paper disks that were applied on the tip of the tongue for 30 s and then spat out. The LMS [89] was also used in this test to rate the perceived intensity of each paper disk. Subjects who rated the PROP disk higher than 67 were classified as STs, those who rated the PROP disk lower than 15 mm were classified as NTs, and all others were categorized as MTs [7]. Four subjects classified as STs based on the three-solution test resulted as MTs when the impregnated paper screening test was used. These four subjects were excluded from the study. 

Since subjects classified as STs by LMS could overestimate the oral stimuli compared to the other taster groups [4], in order to validate ST classification, these subjects were also tested by using the general labeled magnitude scale (gLMS) [90], which expands the top anchor of the scale to include any sensation. The subjects were asked to use the gLMS to rate the heaviness of 6 opaque sand-filled jars (ranging from 235 to 955 g). Heaviness ratings were used to normalize PROP taste intensity ratings as previously described [91]; these subjects still resulted as STs. 

Based on their taster group assignments, 16 subjects were STs (5 M, 11 F), 51 were MTs (24 M, 27 F), and 17 (6 M, 11 F) were NTs. 

#### 2.4.2. Scores for the Five Basic Qualities and Taste Sensitivity Status Determination

The taste strip test (TST; Burghart Messtechnik, Wedel, Germany) [83,84] and the umami test (Burghart Messtechnik, Wedel, Germany) [85] were used to examine taste sensitivity to the four qualities (sweet, sour, salty, bitter) and sensitivity to umami, respectively. The two tests consist of filter paper strips impregnated with 4 concentrations of each taste quality (0.05, 0.1, 0.2, or 0.4 g/mL of sucrose; 0.05, 0.09, 0.165, or 0.3 g/mL of citric acid; 0.016, 0.04, 0.1, or 0.25 g/mL of NaCl; 0.0004, 0.0009, 0.0024, or 0.006 g/mL of quinine hydrochloride; and 0.25, 0.1, 0.04, or 0.016 g/mL of monosodium glutamate, respectively). The subject placed each paper strip on the tongue and identified the taste quality. The correct answers were rated 1; thus, the maximum score for each taste quality was 4, for the four qualities of the TST was 16, and 20 when the evaluation of umami was included (overall TST). A subject was considered normogeusic if they scored ≥ 9, and hypogeusic or ageusic if they scored < 9, as described by Landis et al. [83]. Taste qualities were presented in a pseudo-randomized manner, and concentrations were tasted from the lowest to the highest. Before each stimulation, the subjects rinsed their mouths with spring water.

### 2.5. Papilla Density

The fungiform papillae were identified as described by Melis et al. [15]. The tip of the anterior tongue surface was dried and stained by placing (for 5 s) a disk of filter paper (6 mm diameter) impregnated with a blue food dye (E133, Modecor Italiana, Italy) on the left side of the tongue midline. Then, 3–10 photographs of the stained area were taken in each subject using a Canon EOS D400 (10 megapixels) camera with an EFS 55–250 mm lens. The fungiform papillae were identified from the images by their mushroom shape and very light staining [92]. The number of fungiform papillae in the stained area of each image was established by the consensus of five trained observers. The density/cm^2^ was calculated for each subject.

### 2.6. Molecular Analysis 

DNA was extracted from saliva samples using the QIAamp^®^ DNA Mini Kit (QIAGEN S.r.l., Milan, Italy) according to the manufacturer’s instructions. The concentration of purified DNA was assessed by measurements at an optical density of 260 nm with an Agilent Cary 60 UV–Vis Spectrophotometer (Agilent, Palo Alto, CA, USA).

Subjects were genotyped for three single-nucleotide polymorphisms (SNPs), (*rs713598*, *rs1726866*, and *rs10246939)* of the *TAS2R38* locus, which resulted in three amino acid substitutions (Pro49Ala, Ala262Val, and Val296Ile), and they were also genotyped for the *rs2274333* (A/G) polymorphism of the gustin gene (CA6) that consists of the substitution of Ser90Gly. Molecular analyses were performed using TaqMan SNP Genotyping Assays (C_8876467_10 assay for the *rs713598*, C_9506827_10 assay for the *rs1726866*, C_9506826_10 assay for the *rs10246939*, and C_1739329_1_assay for the *rs2274333*) according to the manufacturer’s specifications (Applied Biosystems by Life Technologies Milan, Italy, Europe BV). Each reaction included two negative controls, three positive controls (one for each genotype), and two replicates. 

Based on molecular analysis at the three SNPs of the *TAS2R38* locus, 20 subjects were PAV homozygous, 43 were heterozygous, and 21 were AVI homozygous. Since rare haplotypes contribute to intermediate sensitivity [93], subjects with rare haplotypes were excluded from the study in order reduce confounding factors. 

In addition, 49 subjects were homozygous AA for the SNP *rs2274333* of the gustin gene, while 29 were heterozygous, and 6 were homozygous GG.

### 2.7. Machine Learning

In order to automatically classify subjects as belonging to a PROP taster category (ST, MT, or NT), we used supervised learning (SL), which involves a type of algorithm that uses labeled datasets (previously recorded from subjects) to train models in order to make predictions on new samples. In our work, the labeled dataset comprised different independent variables (features) and a corresponding dependent variable for each sample (PROP taster category). The dataset was randomly divided into 75% training data and 25% test data. During training, the algorithm searches the data for patterns that correlate with each category. After training, the SL algorithm takes new unknown inputs (in the test data) to determine to which PROP taster categories they must be assigned. We used the following algorithms: logistic regression, decision trees, random forests, *k*-nearest neighbors (*k*-NN) (version: scikit-learn 0.23.2; website: https://scikit-learn.org (accessed on 29 October 2021)), and the CatBoost classifi-er (version: catboost 0.24.3; software: https://arxiv.org/abs/1706.09516 (accessed on 29 October 2021); website: https://catboost.ai/ (accessed on 29 October 2021)). The latter is a new gradient-boosting algorithm that supports categorical features as inputs [94], and is known for its advantages in handling small datasets [94,95,96].

Learning curve graphs, which are generally used as a diagnostic tool to assess the incremental performance of a model as the controlled parameter changes, can also be employed to estimate the required dataset size. Supplementary Figure S1 shows how the performance of the SL model used in our approach initially increases with the increase in the size of the training dataset. Subsequently, at dataset size values corresponding to the ones used in this work (*n* = 84), the performance of the model saturates, and adding more data does not lead to a significant increase in the performance.

Metrics of evaluations—such as the accuracy (1), precision (2), recall (3), F1-score (4), receiver operating characteristic (ROC) curve, and area under the curve (AUC)—were used to evaluate the training and performance of the SL algorithms.

(1) $$\mathrm{Accuracy}=\frac{\mathrm{true}\;\mathrm{positives}+\mathrm{true}\;\mathrm{negatives}}{\mathrm{all}\;\mathrm{samples}}$$

(2) $$\mathrm{Precision}=\frac{\mathrm{true}\;\mathrm{positives}}{\;\mathrm{true}\;\mathrm{positives}+\mathrm{false}\;\mathrm{positives}}$$

(3) $$\mathrm{Recall}=\frac{\mathrm{true}\;\mathrm{positives}}{\mathrm{true}\;\mathrm{positives}+\mathrm{false}\;\mathrm{negatives}}$$

(4) $$F1-\mathrm{score}=2\;\times \;\frac{\mathrm{precision}\;\times \;\mathrm{recall}}{\mathrm{precision}+\mathrm{recall}}$$

The ROC curve shows a binary or multiclassification classifier’s diagnostic ability as its discrimination threshold is varied. The ROC curve is constructed by plotting the true positive rate (TPR) in function of the false positive rate (FPR) at different threshold settings. The AUC is used in classification analysis to determine which of the used models predicts the most suitable category; a higher AUC indicates a better classifier. Values of the AUC lower than 0.5 indicate that the model has no capacity to discriminate, while a classifier is perfect when the AUC is 1. The micro-average and the macro-average are parameters to evaluate the AUC; the micro-average combines the contributions of all categories to compute the average metric, while the macro-average computes the metric independently for each category, and then makes the average (hence treating all categories equally). In a multiclass classification, micro-average is preferable when there are imbalanced categories. 

The SL algorithms may cause problems of overfitting and underfitting. Overfitting occurs if the model has a high accuracy score on training data but a low score on test data, while underfitting arises if the model has a low accuracy score on both training data and test data. To reduce the possibility of overfitting, we applied cross-validation (as described below), which lowers the number of independent features by removing all non-significant and correlated features from the dataset, and by increasing regularization parameters of the SL algorithms. We did not find any problems of underfitting. 

#### 2.7.1. Data Processing Operations

**Figure 2:**
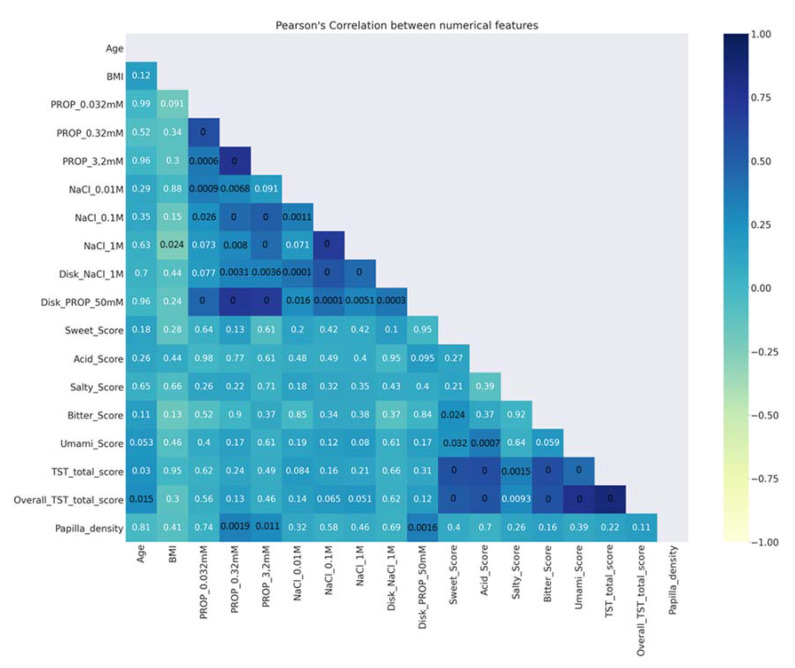
Linear correlation analysis between the numerical features in the dataset. The bar color on the right-hand side on the *Y*-axis represents the value of linear correlation between features, with −1 indicating total negative linear correlation, 0 indicating no linear correlation, and 1 indicating total positive linear correlation. *p*-Values are indicated inside each square: significant values in black; non-significant values in white.

Before applying SL algorithms, the following data processing operations were performed:

1. Description of data analysis: A Pearson’s (r) coefficient analysis [97] was made to verify the correlations between numerical features. Figure 2 shows that the ratings determined with the solutions in the three-solution tests, as well as those determined with impregnated paper disks, were strongly correlated with one another (*r* > 0.43; *p* < 0.001). Moreover, a strong correlation was found between papilla density and the ratings of PROP paper disks (*r* = 0.34; *p* = 0.0016). In addition, the scores of the TST and the overall TST were strongly correlated with the scores of sweet, acid, bitter, and umami (*r* > 0.43; *p* < 0.001). The *p*-values of the significant correlations are shown in black (Figure 2);
2. Preprocessing the data—This operation includes the handling of missing values: (1) removal of the columns/rows with more than 60% missing values; (2) estimation of the missing values (when less than 60%) with the mean or median value of the column; and (3) elimination of the duplicated values from the dataset. After analysis of the dataset, we had to eliminate four variables (sucrose threshold, and intensity ratings of three suprathreshold solutions of sucrose). In addition, in six rows in which the BMI value was lacking, it was estimated by the mean values of the column;
3. Processing the features requires transforming the content of the dataset into a language readable to be processed by an algorithm. This operation includes one-hot encoding (encoding categorical data into numerical data) and normalization of the numerical data (through transforming an actual range of numerical values into a standard range of values between 0 and 1). Finally, the synthetic minority oversampling technique (SMOTE) [98] permits the balancing of the numbers of PROP taster status categories.

#### 2.7.2. Principal Component Analysis

Principal component analysis (PCA) is a method that is used to reduce the dimensionality of datasets, by transforming a set of variables into a smaller one while preserving as much information as possible. We used PCA for the visualization task, by transforming our training data of 34 features into a two-dimension dataset. Since the ST and NT categories have an unequal size of data with respect to the MT category, PCA was performed before and after oversampling of the dataset, including the synthetic samples generated by SMOTE. 

#### 2.7.3. Model Training

After processing of the data, we used cross-validation [99] and compared the following five models: logistic regression, gradient boosting, decision trees, random forests, and CatBoost.

Cross-Validation [99] is a technique for statistical evaluation of ML models by training them on data subsets and assessing them on the corresponding data subset. The algorithm we used to apply the cross-validation was 3-fold cross-validation, which shuffles and divides data into two groups: one group (25% of data) as the test data, and the other (75% of data) as the training data. This procedure was repeated three times, using different subsets each time. Therefore, each sample was used once in the test set and k − 1 times in the training set. In each turn, SMOTE was used to increase the amount of training data in the minority categories. 

#### 2.7.4. Hyperparameter Tuning 

Hyperparameter tuning [100] indicates the automatic optimization of the hyperparameters of an SL model. This process is significant because it measures the overall behavior of an SL model. Among different hyperparameters, we used the grid search algorithm [101] which, by examining every possible combination of each set of hyperparameters, allowed us to find the best hyperparameters for our classifiers. Once the process was completed and we had obtained the best hyperparameters for each model, the models had been evaluated by means of the metrics already listed above.

#### 2.7.5. Model Explainability and Feature Importance 

Explainable SL refers to the tools and techniques that can be employed to interpret any black-box SL model. The tool that we used was Shapley Additive Explanations (SHAP) [102], which is a game-theoretical method to interpret any SL model’s output; it returns a summary plot of SHAP that links feature importance with feature effects. Each point on the summary plot is a Shapley value for a feature and an instance. 

### 2.8. Statistical Analyses

Fisher’s method (Genepop software version 4.7.5; available on the web at the link: https://genepop.curtin.edu.au/genepop_op3.html (accessed on 29 October 2021)) [103] was used to test *TAS2R38* and *gustin* gene genotype distribution according to PROP taster status. One-way ANOVA was used to compare differences in age, BMI, papilla density, and taste scores among STs, MTs, and NTs. Statistical analyses were conducted using STATISTICA for WINDOWS (version 7; StatSoft Inc., Tulsa, OK, USA). *p*-Values < 0.05 were considered significant.

## 3. Results

Demographic, clinical, morphological, and sensory features, along with the genotype distribution of the *TAS2R38* and *gustin* genes determined in the overall sample and according to PROP taster status, are shown in Table 1. One-way ANOVA showed that the density of fungiform papillae varies with PROP taster status (F_[2,81]_ = 6.802; *p* = 0.0019). STs had a higher papilla density than MTs (*p* = 0.041; Fisher’s LDS test), who showed higher density than NTs (*p* = 0.016; Fisher’s LDS test). The *TAS2R38* SNPs were associated with PROP taster status based on genotype distribution (χ2 = 31.884; *p* < 0.0001; Fisher’s test). Pairwise comparisons discriminated all groups from one another (χ2 > 13.66; *p* < 0.0011; Fisher’s test). The genotype AVI/AVI was more frequent (88.23%) in NTs, PAV/AVI was more frequent in MTs (68.63%), and PAV/PAV was more frequent in STs (65.50%). No differences related to PROP taster status in terms of age, BMI, gender, smoking status, genotype distribution of gustin locus, or taste scores were found (*p* > 0.05).

**Figure 3:**
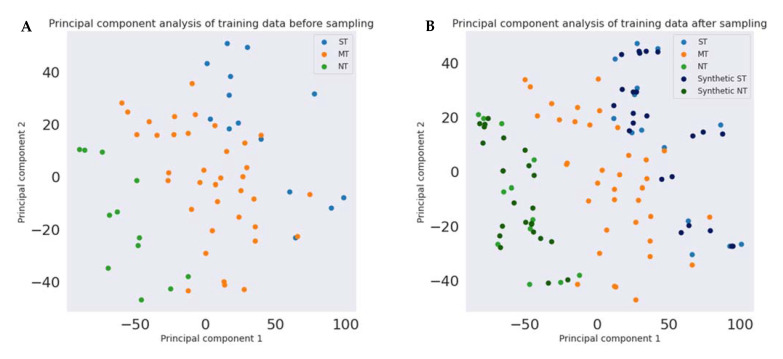
Scatterplots of the NT (green circles), ST (blue circles), and MT (orange circles) samples of combined features derived via PCA before (**A**) and after (**B**) SMOTE. Synthetic samples generated by SMOTE in minority categories are shown (dark blue: ST; dark green: NT). The *X*- and *Y*-axes in each graph represent combinations of all features used in the experiments.

Figure 3 shows two scatterplots obtained via PCA, in which a reduction in the dimensionality of the training set was performed before (A) and after oversampling data (B); the latter includes the synthetic samples generated by SMOTE. The combination of features obtained via PCA visibly shows the differences between the three PROP taster categories.

The values of the accuracy, precision, and F1-score (metrics of evaluation used to assess the training and performance of the SL algorithms) showed that the CatBoost algorithm enabled us to achieve objective PROP taster status identification with a high precision (97%), high recall (95%), and an F1-score of 96% (Table 2). Other algorithms (i.e., logistic regression, gradient boosting, decision trees, and random forests) also achieved objective PROP taster status identification, but with lower values of precision, recall, and F1-score compared to CatBoost (Table 2).

**Figure 4:**
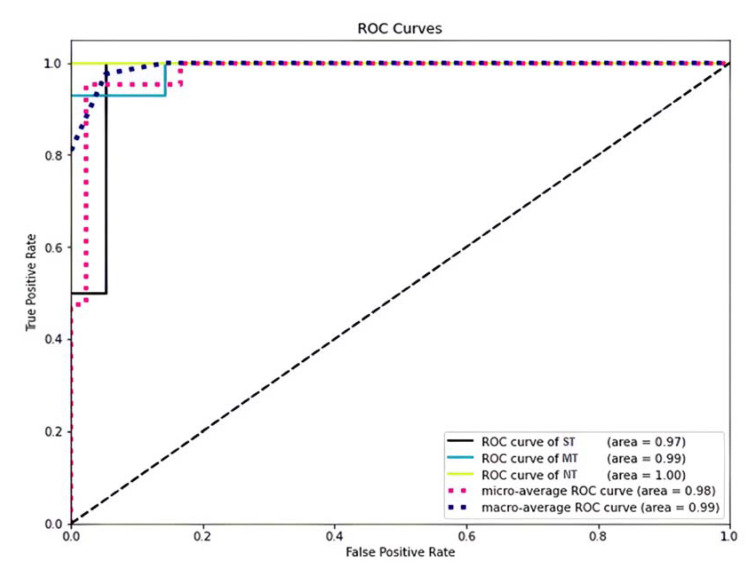
The ROC curve and the AUC of the CatBoost model. The ROC curve is made by plotting the rate of true positives as a function of the rate of false positives. The black line represents the correct predictions of the STs, the light blue line represents the correct predictions of the MTs, and the yellow line represents the correct predictions of the NTs. Micro- and macro-average ROC curves are also represented by the dotted pink line and the dark blue dotted line, respectively.

Figure 4 shows the ROC curve and AUC of the three PROP taster categories obtained via the CatBoost model. In particular, the ROC curve shows that the corrected predictions made by the model were 97%, 100%, and 99% for the ST, NT, and MT categories, respectively. In addition, the AUC of this classifier showed that the average margin of error, which was represented by the micro-average, was 98% for all categories. The macro-average was 99% for all categories, but this was not significant because we had unbalanced data in the test set. In addition, Supplementary Figure S2 shows an alluvial plot representing the changes in network structure over subject groups identified by different methods (i.e., *TAS2R38* genotypes, PROP taster categories, and SL discrimination).

**Figure 5:**
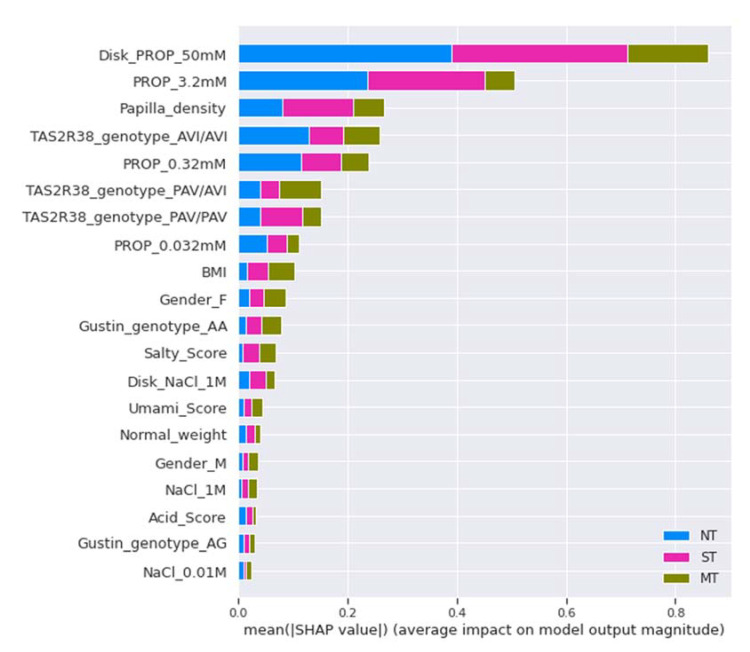
Feature importance of the CatBoost classifier in the training set. The *X*-axis represents the average impact on the model output, while the *Y*-axis represents the order of importance of the features in the training set to understanding the categories of each PROP taster status.

The CatBoost classifier allowed us to determine the order of importance of the biological features in facilitating the learning of the model to identify the three PROP taster status categories (Figure 5). Specifically, in the figure, blue indicates the importance of features for training the NT category, pink for training the ST category, and green for training the MT category. The intensity rating for PROP paper disks (50 mM) was the most important feature in the training set, followed in order of importance—from second to the seventh—by intensity rating for the PROP solution (3.2 mM), fungiform papilla density, AVI/AVI genotype, intensity rating for PROP solution (0.32 mM), PAV/AVI genotype, and PAV/PAV genotype. It is interesting to note that, among the scores given to taste qualities, those for salty and umami were the most significant in facilitating the learning of the model. Furthermore, gender was a significant feature in facilitating training, with the female gender (10th in order of importance) more significant than the male gender (16th in order of importance).

The SHAP algorithm allowed us to obtain an overview of the most important features for the model, and how they impact it to make a prediction.

**Figure 6:**
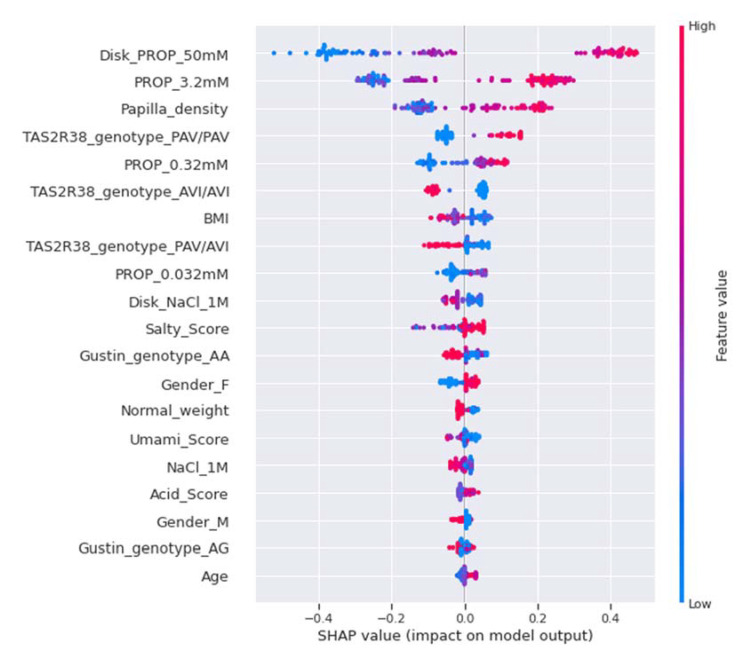
SHAP summary plot of the ST category. The left-hand side of the *Y*-axis represents the descending order of importance of the ST category features; the *X*-axis represents the impact of the SHAP value on the output model. The color represents the feature value: high values have a pink color, while low values have a blue one.

The SHAP summary plot for the ST category is shown in Figure 6; the descending order of the feature importance of the ST category (from the most important at the top to the least important at the bottom) is shown on the left-hand side of the *Y*-axis. Conversely, the position of the value on the *X*-axis shows whether the feature is associated with a higher or lower prediction score for the ST category. Specifically, the SHAP summary plot for the ST category highlights that the intensity rating for PROP paper disks (50 mM) was the most important feature for the model, and high estimated values (pink) were strongly and positively correlated with the ST category. The intensity rating for the PROP solutions (3.2 mM) was the second in order of importance for the ST category, and high estimated values (pink) were positively correlated with this category. Papilla density was the third feature in order of importance for this category, and high estimated values (pink) were positively correlated with it. Low and medium values of these three features (blue and violet, respectively) pushed the model prediction towards other categories. PAV/PAV genotype was the fourth most important feature, which was positively correlated with the ST category, and was more important than the AVI/AVI genotype for the prediction of the ST category. The intensity rating for the PROP solutions (0.32 mM) was the fifth in order of importance, and high estimated values (pink) were positively correlated with the ST category, while low and medium values (blue and violet) pushed the model prediction towards other categories. Furthermore, salty and umami scores were significant predictive features, and the high and low estimated values, respectively, had a moderate impact on ST prediction. Female gender was the 13th most important feature, and was correlated moderately and positively with the ST category, while male gender was less important, and was moderately and negatively correlated with this category.

**Figure 7:**
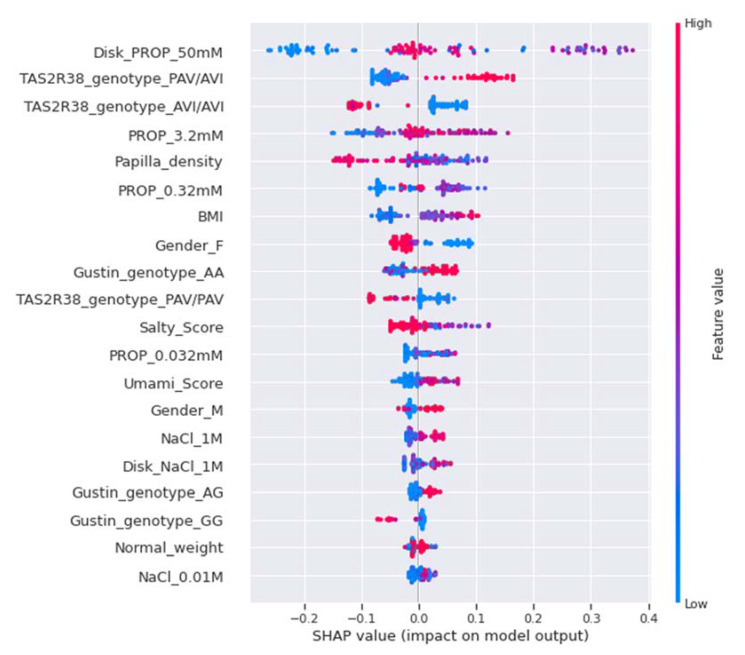
SHAP summary plot of the MT category. The left-hand side of the *Y*-axis represents the descending order of importance of the MT category features; the *X*-axis represents the impact of the SHAP value on the output model. The color represents the feature value: high values have a pink color, while low values have a blue one.

The SHAP summary plot for the MT category is shown in Figure 7. For this category, the intensity rating for PROP paper disks (50 mM) was also the most important feature for the model; however, medium estimated values (violet) were strongly and positively correlated with MT category, while high and low estimated values (pink and blue) pushed the model prediction towards other categories. It is interesting to note that PAV/AVI genotype was the second feature in order of importance, and was positively correlated with the MT category, while AVI/AVI genotype was the third feature, and was negatively correlated with this category. The intensity rating for the PROP solutions (3.2 mM) was the fourth feature, and medium estimated values (violet) were positively correlated with this category. The fifth feature in order of importance was papilla density, and high estimated values were negatively correlated with the MT category. The intensity rating for the PROP solutions (0.32 mM) was the sixth feature in order of importance, and medium estimated values (violet) were positively correlated with this category. The salty and umami scores were significant features, and medium estimated values prompted the model to make an MT prediction. Female gender was the 8th most important feature, and was negatively correlated with the MT category, while male gender was less important, and was moderately and positively correlated with this category.

**Figure 8:**
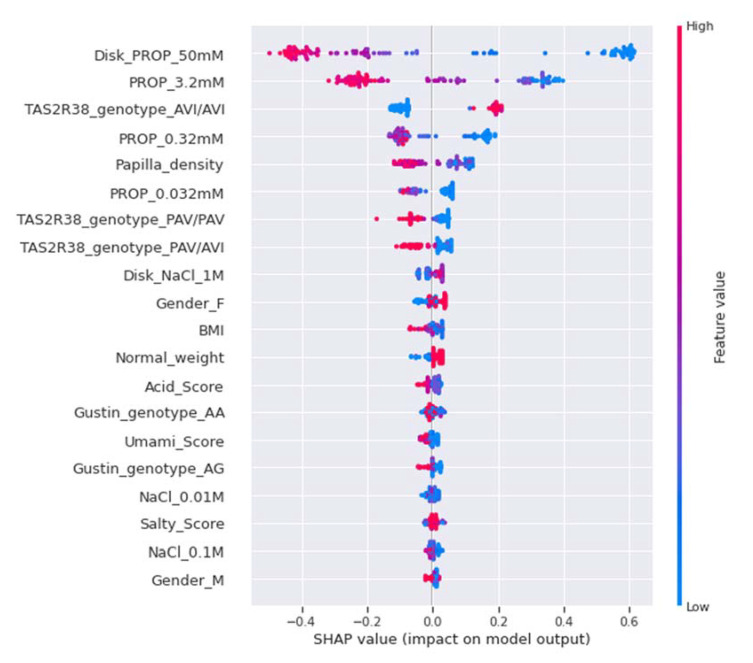
SHAP summary plot of the NT category. The left-hand side of the *Y*-axis represents the descending order of importance of the NT category features; the *X*-axis represents the impact of the SHAP value on the output model. The color represents the feature value: high values have a pink color, while low values have a blue one.

The SHAP summary plot for the NT category is shown in Figure 8. In this case, the intensity ratings for PROP paper disks (50 mM) and the PROP solutions (3.2 mM) were the first and the second features in order of importance, respectively, and low estimated values (blue) were strongly and positively correlated with the NT category. AVI/AVI genotype was the third most important feature and was positively correlated with this category. The intensity rating for PROP solution (0.32 mM) was the fourth most important feature, and low estimated values (blue) were positively correlated with the NT category. Low estimated values of papilla density, which was the fifth most important feature, were positively correlated with this category, while high values were negatively correlated with it. Sour and umami scores were significant features, and medium and low estimated values, respectively, moderately pushed the model toward an NT prediction. The female gender was the 10th most important feature and was moderately and positively correlated with the NT category, while the male gender was the least significant feature, and was moderately and negatively correlated with this category.

## 4. Discussion

The goal of this work was to build an ML model capable of automatically predicting the PROP taster status with high precision, and to deeply understand the importance and the impact of the specific biological features of 84 subjects aged from 18 to 40 y, which were presented in the data model.

We used SL that operates a set of algorithms and computational statistics to learn from data without being explicitly computed and creates models that can make predictions on new samples. In our approach, we used different algorithms, such as logistic regression, decision trees, random forests, *k*-nearest neighbors (*k*-NN), and the CatBoost classifier. Our results showed that the CatBoost classifier was the best model for the automatic classification of the PROP taster status, as shown by the high metric values of accuracy, precision, and F1-score determined with this model. The analysis of the ROC curves and AUCs of the three PROP taster categories confirmed that the CatBoost classification model is the best model that can be used on our data; it gave the best ROC curve, showing very small error scores (3% error for the ST category predictions, 0% for predicting NT, and just 1% error for identifying MT). In addition, this model does not overfit, because it achieved approximately the same prediction scores on the training data and on the test data. The fact that CatBoost was the best model for our data is not surprising; in fact, CatBoost is an algorithm for gradient boosting on decision trees and delivers the best results when a dataset has a lot of categorical features. Moreover, it is broadly known that CatBoost can be applied across a wide range of areas and to a variety of problems [94]. Nevertheless, our results showed that the other algorithms could also achieve objective PROP taster status identification, but with a lower precision as compared to CatBoost.

Furthermore, the CatBoost classifier allowed us to obtain the order of importance of the biological features used as a dataset in facilitating the learning of the model, aimed at understanding the difference between the three PROP taster status categories. The analysis showed that the intensity rating for PROP paper disks (50 mM) was the most important feature for the model. This result is of great importance, as it indicates that the impregnated paper screening test [7], which is the simplest and fastest psychophysical approach, provides the best features in the training of the model to make predictions on the PROP taster status of new samples. This suggests the choice of this test in the planning of psychophysical experiments for the subjects’ PROP taster status classification. Our results also highlight the importance of the use of the three-solution test [5], which provided a biological feature—the intensity rating for PROP solution (3.2 mM)—which was the second feature in order of importance in the training model.

Our results also showed that the density of the fungiform papillae was a significant feature (the third in order of importance) for the training model to learn to distinguish between the PROP taster categories and was particularly effective in making the ST prediction. In fact, the SHAP algorithm showed that high estimated values of papilla density were strongly and positively correlated with the ST category. These results are consistent with data showing that STs have a higher density of fungiform taste papillae, as compared to the other PROP taster categories [6,15,16,18,78,81].

Our results also showed that the *TAS2R38* locus provides significant features for training the model. However, although the molecular analysis of the locus is an objective measure, its importance in the training system was lower than that of the features obtained by psychophysical tests. This is unsurprising, since the *TAS2R38* genotypes do not completely explain the oral sensory differences between MTs and STs [8], and a considerable genotypic overlap exists between these two groups [10,13,104]. These considerations suggest a more effective use of psychophysical approaches in studying taste sensitivity, rather than the determination of genotype alone for specific receptors.

According to findings showing associations between perceptions for the five taste qualities [19,20,21,23,25,26,48,49,50,88] and for PROP, our results showed that the scores that the subjects gave to tastes (that are not mediated by specific receptors) were significant features in facilitating the learning of the model to differentiate categories of PROP tasters.

Furthermore, gender was a significant feature, with the female gender—which has been shown to have a higher sensitivity to the PROP taste [6,42,43]—being more important to training the model than the male gender.

The SHAP approach allowed us to produce a high-precision explanation for the predictions that the model makes for each category of the PROP taster status. The SHAP approach confirmed the importance of the features used in the training step, and provided an explanation of how it uses each feature. Specifically, the SHAP results showed that high values of the intensity rating for PROP paper disks (50 mM) strongly pushed the model to make an ST prediction. Similarly, high estimated values for the intensity rating of PROP solution (3.2 mM), as well as those of papilla density or the PAV/PAV genotype, pushed the model towards the ST prediction. On the other hand, the SHAP approach showed that medium estimated values of the intensity rating for the PROP paper disks (50 mM) powerfully pushed the model to make an MT prediction. Accordingly, the PAV/AVI genotype had an impact favorable to the prediction of the MT category, while the AVI/AVI genotype was negatively correlated with this category. Finally, SHAP results showed that low estimated values for the intensity ratings for PROP paper disks (50 mM) and PROP solutions (3.2 mM) were directly correlated with the NT category, while the AVI/AVI genotype also pushed the model to make an NT prediction; in addition, papilla density was negatively correlated with this category. It is interesting to note that the salty and umami scores were both qualities that significantly impacted the model to make an ST or MT prediction, while the sour perception moderately pushed the model toward an NT prediction; future studies may investigate this phenomenon. In addition, according to data showing that the female gender has a higher PROP taste sensitivity than the male gender [6,42,43], the SHAP approach showed that the female gender was more important in impacting the model to make a prediction. Specifically, the female gender was positively and moderately correlated with the ST and NT categories and negatively correlated with the MT category. Conversely, the male gender was moderately and negatively correlated with ST and NT, while positively correlated with the MT category.

All of these results indicate that the classification model CatBoost used approximately the same reasoning as a biology expert to classify individuals and assign them the correct PROP taster category.

The ML methods require big data to fit the algorithms, and bias is expected to be larger for smaller datasets. However, depending on the problem domain, dataset size is not necessarily a barrier to a high-performing model, since the average performance of classifiers reached 99% on some small datasets. Althnian et al. [105] showed that the overall performance of SL classifiers depends on how much a dataset represents the original distribution rather than its size. Indeed, in addition to depending on the size of the samples, bias can also depend on other properties of the dataset, e.g., the dependency between the features and the target [94]. Based on these considerations, since we have a small sample size, we devoted more effort to selecting only the relevant features, removing outliers from the data, handling missing data, and oversampling the training set in order to balance the class distribution. Additionally, gradient-boosting decision trees (GBDTs) in general—and CatBoost in particular—are known for their advantages in handling small datasets; they perform better than the other types of models on a small dataset [94,95,96], while regulating the multiple parameters of CatBoost helps us to avoid the overfitting of the model. To reduce the bias and the variability, we conducted multiple rounds of cross-validation (K-fold, where k equals 3) with distinct subsets from the same data. The F1-score results from these multiple rounds were very close, ensuring that the model performs well on the full dataset. Therefore, in our approach, the preparation and processing of the dataset, as well as the analysis of the dataset and the definition of correlations between parameters, were fundamental steps that allowed us to scale up the dataset quality and attain better results. We found strong correlations of the rating values of PROP and NaCl stimuli with one another, as well as between fungiform papilla density and PROP rating, and the whole taste perception and perceptions of sweet, acid, bitter, and umami. The sensitivity to the bitterness of PROP varied considerably between PROP categories: NT individuals perceived PROP as low intensity, MTs as medium intensity, and STs as high intensity. We also found a strong association between the genotype of the *TAS2R38* and PROP taster status, since individuals who had the AVI/AVI genotype could not be ST, while those who had the PAV/PAV genotype could not be NT. On the other hand, the two genotypes PAV/PAV and PAV/AVI could both be determined in MT and ST individuals. We also found that the three PROP taster categories were clearly distinguished after feature scaling.

## 5. Conclusions

In conclusion, our results show that the proposed SL approach is a reliable tool for the automatic classification of PROP taster status, through fully automatic processing, by including biological features of subjects that are normally used for the classification of subjects as belonging to the PROP taster categories in the psychophysical methods [5,7], or that have been associated with PROP taster status in physiological studies [3,4,6,10,11,15,80,81]. The proposed SL approach allowed us to achieve the high-precision automatic classification of PROP taster status of subjects, which could make this process immediate and scalable. Furthermore, this method gave us the possibility to understand which features are the most significant as predictive factors to make a precise distinction between ST, MT, and NT subjects, and identify the parametric patterns and correlations. In this way, the SL approach allowed us to identify biomarkers or combinations of biomarkers among the considered biological features, to be applied to large epidemiological studies instead of time-consuming tests.

In this study, we were able to automatically identify the PROP phenotypes of 84 subjects aged from 18 to 40 y, with high precision, and in future studies this method could be applied for the identification of PROP genotypes, thus reducing the costs and time of molecular analysis of the *TAS2R38* locus. The SL model, or other types of ML (appropriate for unstructured data, such as the density of the fungiform papilla from pictures of the tongue), may be extended to physiological studies on taste, with applications ranging from basic science and medicine to food tasting evaluations.

## Figures and Tables

**Table 1:** Demographic, clinical, morphological, genetic, and sensory features of subjects according to PROP taster status.

Features	Overall	ST (*n* = 16)	MT (*n* = 51)	NT (*n* = 17)
Age (year)	25.07 ± 0.46	24.94 ± 1.07	24.95 ± 0.60	25.59 ± 1.03
BMI (kg/m^2^)	21.82 ± 0.36	20.83 ± 0.82	22.27 ± 0.46	21.41 ± 0.79
Papilla density/cm^2^	30.63 ± 1.58	39.04 ± 3.39 ^a^	30.98 ± 1.90 ^b^	21.68 ± 3.29 ^c^
Male/female (*n*)	35/49	5/11	24/27	6/11
Smokers/non-smokers (*n*)	19/65	3/13	13/38	3/14
Genotypes				
*TAS2R38*				
PP/PA/AA (*n*)	20/43/21	10/6/0 ^x^	10/35/6 ^y^	0/2/15 ^z^
Gustin gene				
AA/AG/GG (*n*)	49/29/6	9/4/3	27/22/2	13/3/1
Taste scores				
Sweet	3.43 ± 0.07	3.37 ± 0.17	3.43 ± 0.09	3.47 ± 0.16
Salty	3.57 ± 0.08	3.81 ± 0.17	3.51 ± 0.10	3.53 ± 0.17
Sour	2.38 ± 0.10	2.44 ± 0.24	2.41 ± 0.13	2.23 ± 0.23
Bitter	3.21 ± 0.11	3.50 ± 0.26	3.15 ± 0.15	3.11 ± 0.25
Umami	1.32 ± 0.16	1.12 ± 0.38	1.43 ± 0.21	1.17 ± 0.37
TST	12.79 ± 0.22	13.19 ± 0.50	12.80 ± 0.28	12.41 ± 0.49
Overall TST	13.92 ± 0.31	14.25 ± 0.72	13.94 ± 0.40	13.53 ± 0.70

Values are means ± SE or number of subjects. Significant differences in papilla density are indicated by the letters ^a^, ^b^, and ^c^ (*p* ≤ 0.041; LSD test subsequent to one-way ANOVA), while differences in *TAS2R38* genotype distribution are indicated by the letters ^x^, ^y^, and ^z^ (*p* < 0.0001; Fisher’s method). BMI: body mass index; PP: PAV/PAV; PA: PAV/AVI; AA: AVI/AVI; TST: total taste score; Overall TST: overall total taste score.

**Table 2:** Results of metrics to evaluate each classifier model.

Classifiers	Precision	Recall	F1-Score
Logistic regression	83%	81%	81%
Gradient boosting	90%	86%	87%
Decision trees	92%	90%	91%
Random forests	96%	95%	95%
CatBoost	97%	95%	96%

## Data Availability

The data presented in this study are available on request from the corresponding author. The data are not publicly available, in accordance with consent provided by participants on the use of confidential data.

## Supplementary Materials

The following are available online at https://www.mdpi.com/article/10.3390/nu14020252/s1, Figure S1: Learning curve graph employed to estimate the performance of the SL as dataset size increases., Figure S2: Alluvial plot illustrates the changes in the composition of the clusters (*TAS2R38* genotypes, PROP taster categories and SL discrimination).
