# Supplementary material for: Automated Classification of 6-n-Propylthiouracil Taster Status with Machine Learning
Source: Nutrients. 2022 Jan 7;14(2):252. doi: 10.3390/nu14020252 (PMC8778915; doi:10.3390/nu14020252)
Supplement: Supplementary file 1 [file nutrients-14-00252-s001.zip › nutrients-1470676-supplementary.pdf]

Supplementary material

Automated Classification of 6-n-Propyltiouracil Taster Status with Machine Learning

Lala Chaimae Naciri, Mariano Mastinu, Roberto Crnjar, Iole Tomassini Barbarossa* and Melania Melis

Learning curve graphs, which are generally used as a diagnostic tool to assess the incremental performance of a model as the controlled parameter changes, can also be employed to estimate the required dataset size.

Supplemental Figure S1 shows how the performance of the SL model used in our approach initially increases with the increase of the training dataset size. Successively, around at values of dataset size corresponding to those of this work (n = 84), the performance of the model saturates and adding more data does not lead to a significant increase in the performance.

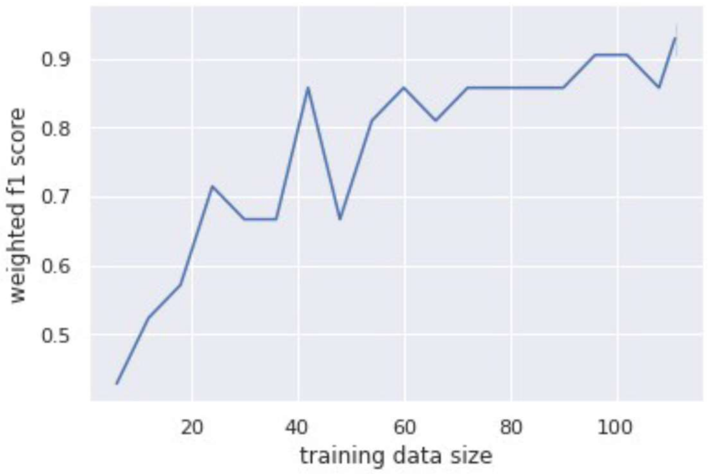

Figure S1. Learning curve graph employed to estimate the performance of the SL as dataset size increases.

Supplemental Figure S2 shows an alluvial plot representing the changes in network structure over subject groups identified by different methods (*TAS2R38* genotypes, PROP taster categories and SL discrimination between PROP taster categories). This diagram visually illustrates that most subjects classified as ST by psychophysical approaches, who had genotype PAV/PAV (*n* = 10) or PAV/AVI (*n* = 6), were assigned to ST category by SL discrimination; most of subjects classified as MT by psychophysical approaches, with a PAV/PAV (*n* = 10), PAV/AVI (*n* = 35) or AVI/AVI (*n* = 6) genotype, were assigned to MT category by SL discrimination; most of subjects classified as NT by psychophysical approaches, with a PAV/AVI (*n* = 2) or AVI/AVI (*n* = 15) genotype, were assigned to NT category by SL discrimination.

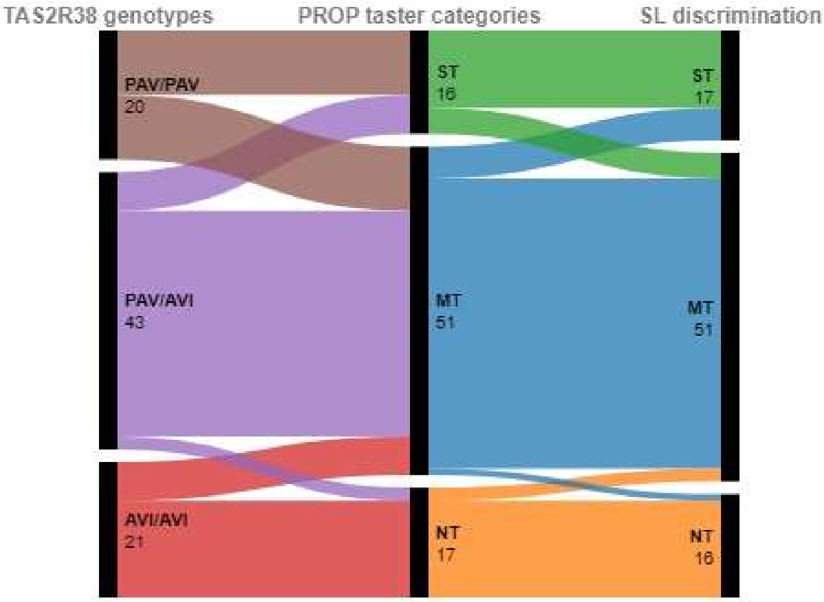

Figure S2. Alluvial plot illustrates the changes in the composition of the clusters (*TAS2R38* genotypes, PROP taster categories and SL discrimination). The height of a stream represents the size of the components contained in the three blocks connected by the stream. Numbers indicate the n of subjects for each cluster.
